# Impact of Extracardiac [^18^F]FDG-PET/CT Findings on Diagnosis and Management in Patients with Suspected Infective Endocarditis

**DOI:** 10.3390/antibiotics15100994

**Published:** 2026-10-07

**Authors:** Jean Regina, Christel H. Kamani, David C. Rotzinger, Nicolas Fourré, Virgile Zimmermann, Nicoleta Ianculescu, Georgios Tzimas, Pierre Monney, Florian Desgranges, Lars Niclauss, Matthias Kirsch, Benoit Guery, John O. Prior, Matthaios Papadimitriou-Olivgeris

**Affiliations:** 1 Department of Internal Medicine, Lausanne University Hospital and University of Lausanne, 1011 Lausanne, Switzerland; jean.regina@chuv.ch; 2 Department of Nuclear Medicine and Molecular Imaging, Lausanne University Hospital and University of Lausanne, 1011 Lausanne, Switzerland; 3 Department of Radiology, Lausanne University Hospital and University of Lausanne, 1011 Lausanne, Switzerland; 4 Infectious Diseases Service, Lausanne University Hospital and University of Lausanne, 1011 Lausanne, Switzerland; 5 Department of Cardiology, Lausanne University Hospital and University of Lausanne, 1011 Lausanne, Switzerland; 6 Department of Cardiac Surgery, Lausanne University Hospital and University of Lausanne, 1011 Lausanne, Switzerland; 7 Infectious Diseases Service, Hospital of Valais and Institute Central des Hôpitaux, 1951 Sion, Switzerland

**Keywords:** infective endocarditis, embolic events, [^18^F]FDG-PET/CT, bone and joint infections, bacteremia, bloodstream infection, vascular graft infection, Duke criteria, source control, *Staphylococcus aureus*

## Abstract

**Background/Objectives:** We aimed to evaluate the incremental diagnostic and clinical impact of extracardiac findings detected by [^18^F]Fluorodeoxyglucose Positron Emission Tomography/Computed Tomography ([^18^F]FDG-PET/CT) in patients with suspected infective endocarditis (IE). **Methods:** This monocentric cohort study included adult patients with bacteremia and suspected IE who underwent [^18^F]FDG-PET/CT. IE diagnosis was based on the 2023 Duke-ISCVID criteria. Extracardiac findings detected by [^18^F]FDG-PET/CT were assessed for their impact on IE diagnostic classification according to the 2023 Duke-ISCVID criteria and on clinical management. **Results:** Among 500 patients with bacteremia and suspected IE who underwent [^18^F]FDG-PET/CT, IE was diagnosed in 211 (42%) episodes. Embolic events were detected in 60 (12%) episodes, with 42 (8%) episodes representing previously unrecognized findings. Two (0.9%) of the 211 IE episodes were reclassified from possible to definite IE according to the 2023 Duke-ISCVID clinical criteria. [^18^F]FDG-PET/CT identified new infectious foci in 31 (6%) episodes (18 bone and joint infections and 13 vascular graft infections). Extracardiac findings on [^18^F]FDG-PET/CT resulted in prolongation of antibiotic treatment in 28 (6%) episodes and in a new indication for source control intervention in 23 (5%) episodes. **Conclusions:** Although [^18^F]FDG-PET/CT identified previously unrecognized embolic events in suspected IE, these findings had limited impact on IE diagnostic classification, supporting a selective rather than systematic use for embolic screening alone. However, its ability to detect occult extracardiac infectious foci may provide clinically relevant added value, particularly in patients with bacteremia of unknown source or vascular grafts.

## 1. Introduction

Embolic events are among the most frequent complications of infective endocarditis (IE), occurring in approximately 20–50% of patients, and are associated with a substantial impact on clinical outcomes [1,2]. In patients with bacteremia, particularly those at high risk for IE, the presence of embolic events raises suspicion of IE and typically prompts a comprehensive diagnostic evaluation [3,4,5]. Embolic phenomena are included as a minor vascular criterion in the Duke diagnostic criteria for IE [5,6], and are incorporated into several prediction scores designed to estimate the likelihood of IE among patients with *Staphylococcus aureus* or enterococcal bacteremia [7,8,9].

The central nervous system is the most frequent site of embolization, followed by abdominal organs [3,4,10,11,12,13]. However, the majority of embolic events are clinically silent. Recent European Society of Cardiology (ESC) guidelines recommend the use of [^18^F]Fluorodeoxyglucose Positron Emission Tomography/Computed Tomography ([^18^F]FDG-PET/CT) not only in patients with suspected IE, in order to detect distant embolic lesions, mycotic aneurysms, and potential portals of entry [5]. Although [^18^F]FDG-PET/CT, thoracoabdominal computed tomography angiography (CTA) and cerebral imaging are proposed for systematic screening of peripheral lesions even in asymptomatic patients [5], prior studies have reported a limited impact of cerebral or thoracoabdominal imaging, including [^18^F]FDG-PET/CT, on improving the diagnostic classification of IE in asymptomatic patients with suspected IE [3,4].

Among patients with suspected IE, the contribution of cardiac [^18^F]FDG-PET/CT findings to diagnostic classification in the presence of prosthetic intracardiac material is well established [14,15]. In contrast, the diagnostic impact of extracardiac findings alone is scarce [16]. Therefore, the present study aimed to assess the usefulness of [^18^F]FDG-PET/CT for the detection of peripheral embolic events in patients with suspected IE, and to evaluate the impact of extracardiac findings on IE diagnosis and clinical management.

## 2. Results

### 2.1. Study Population

**Figure 1:**
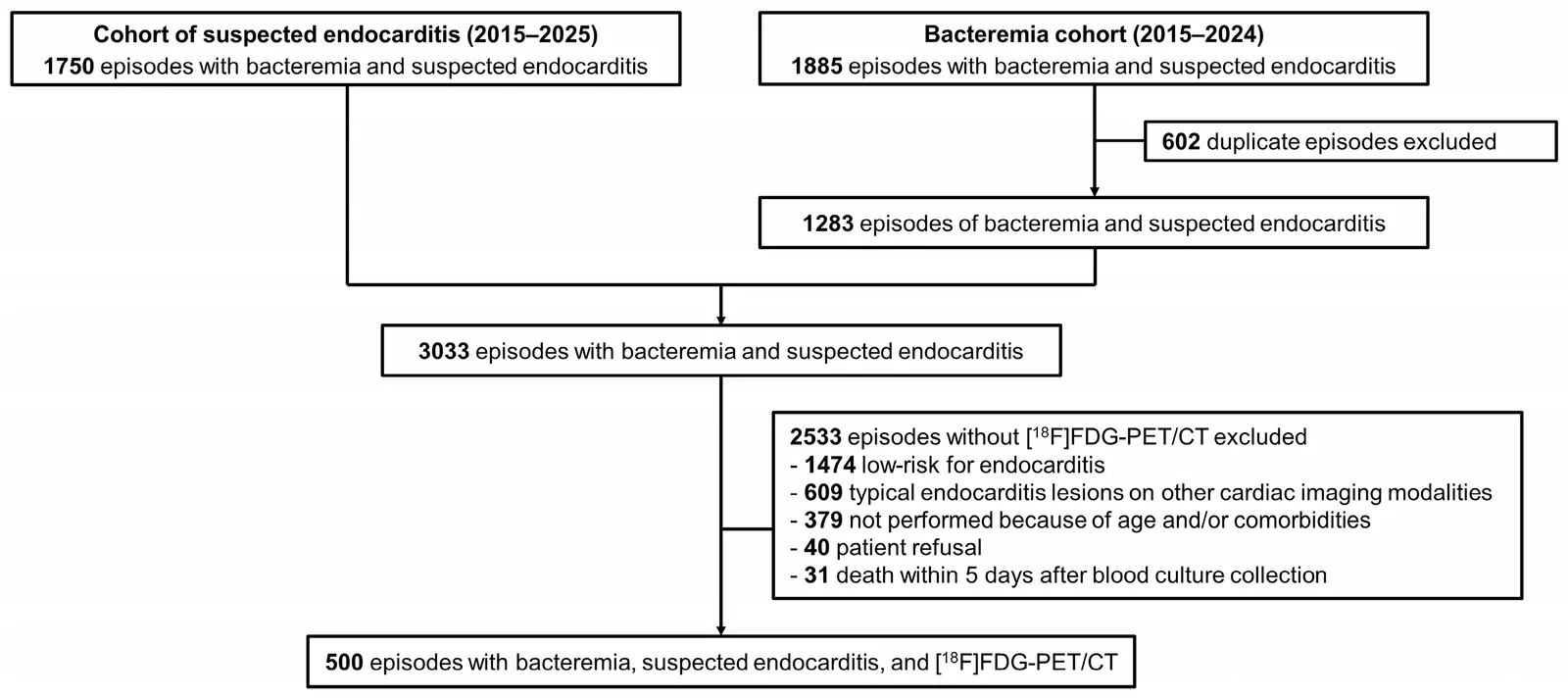
Flowchart of included episodes.

Among 3033 non-duplicate episodes with bacteremia and suspected IE in both cohorts, 500 episodes were associated with bacteremia and included a [^18^F]FDG-PET/CT examination (Figure 1). The most frequently identified pathogen was *S. aureus* (215 episodes; 43%), followed by Enterococci (88; 18%) and Streptococci (80; 16%). ID consultation was provided in 496 (99.2%) episodes.

Transthoracic echocardiography was performed in 457 episodes (91%), transesophageal echocardiography in 316 (63%), and cardiac CTA in 36 (7%). IE was diagnosed by the 2023 Duke-International Society for Cardiovascular Infectious Diseases (ISCVID) criteria in 211 (42%) episodes. Among the 289 episodes in which IE was excluded, the most frequent alternative diagnosis was bone and joint infections (115; 23%), followed by catheter-related bacteremia (94; 19%) (Table 1).

### 2.2. [^18^F]FDG-PET/CT

The median time between the first positive blood culture and the [^18^F]FDG-PET/CT examination was 8 days (interquartile range; IQR: 5–11). Adequate suppression of physiological myocardial glucose uptake was achieved in 406 episodes (81%). Prior to [^18^F]FDG-PET/CT, thoracoabdominal CT and spine MRI had been performed in 245 (49%) and 46 (9%) episodes, respectively. The site of infection was unknown prior to [^18^F]FDG-PET/CT in 176 (35%) episodes. The [^18^F]FDG-PET/CT was performed to assess cardiac lesions in the presence of prosthetic cardiac material and to detect embolic events in 257 (51%) episodes, while in 243 (49%) episodes it was performed solely to detect embolic events or metastatic site of infection. Abnormal cardiac metabolic activity was detected in 106 (21%) episodes.

### 2.3. Embolic Events Detection

Embolic events were identified by [^18^F]FDG-PET/CT in 60 (12%) episodes (Table 1); 42 (8%) episodes represented previously unrecognized embolic events. Among the 255 episodes without prior thoracoabdominal CT, embolic events had been identified in 18 (7%). Among the 245 episodes with prior thoracoabdominal CT, embolic events had been identified in 22 (9%) by thoracoabdominal CT, all of which were confirmed by subsequent [^18^F]FDG-PET/CT (Table 2). Furthermore, [^18^F]FDG-PET/CT provided an incremental diagnostic yield by identifying additional embolic events in 24 (10%) episodes.

### 2.4. Impact on Diagnostic Classification

**Figure 2:**
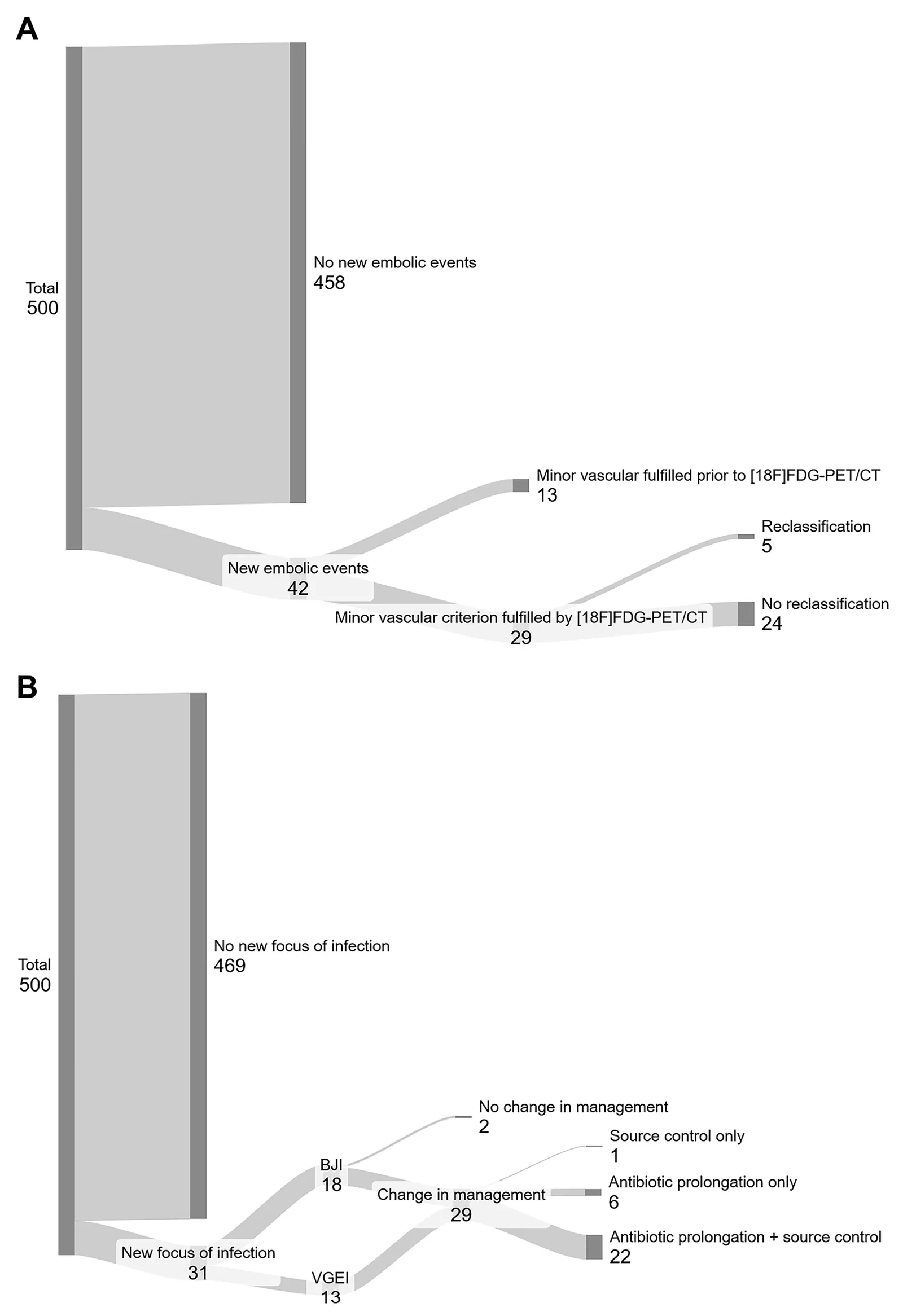
Impact of [^18^F]FDG-PET/CT for (**A**) embolic event detection and impact on diagnostic classification based on 2023 Duke-ISCVID clinical criteria, and (**B**) detection of new infectious foci other than infective endocarditis and their impact on clinical management (prolongation of antibiotic treatment duration and/or new indication for source control intervention) in patients with suspected infective endocarditis and bacteremia. [^18^F]FDG-PET/CT: [^18^F]Fluorodeoxyglucose Positron Emission Tomography/Computed Tomography; ISCVID: International Society for Cardiovascular Infectious Diseases.

Among the 211 IE episodes, 56 (27%) fulfilled the minor vascular phenomena criterion according to the 2023 Duke-ISCVID criteria prior to the performance of [^18^F]FDG-PET/CT, with an additional 21 (10%) episodes meeting this criterion based on [^18^F]FDG-PET/CT findings. This led to reclassification of two (0.9%) episodes from possible to definite IE according to the 2023 Duke-ISCVID clinical criteria (Figure 2A). When the 2015 Duke-ESC clinical criteria were applied, two (0.9%) episodes among the 211 IE cases were also reclassified from possible to definite IE (Table S1).

### 2.5. New Infectious Foci and Impact on Clinical Management

New infectious foci other than IE were identified by [^18^F]FDG-PET/CT in 31 (6%) episodes (18 bone and joint infections and 13 vascular graft infections). These findings resulted in prolongation of antibiotic treatment in 28 (6%) episodes and in a new indication for source control intervention in 23 (5%) episodes (Table S2). Figure 2B summarizes the impact of [^18^F]FDG-PET/CT for the detection of previously unrecognized infectious foci and their impact on patient management. Among the 255 episodes without prior thoracoabdominal CT, [^18^F]FDG-PET/CT identified new infectious foci other than IE in 13 (5%) episodes (10 bone and joint infections and 3 vascular graft infections). These findings resulted in prolongation of antibiotic treatment in 12 (5%) episodes and in a new indication for source control intervention in 8 (3%) episodes.

## 3. Discussion

In this large cohort of patients with bacteremia and suspected IE, the use of [^18^F]FDG-PET/CT led to the detection of new embolic events in 8% of episodes, with no meaningful impact on the diagnosis of IE. However, in 6% of episodes, previously unrecognized findings led to changes in clinical management, including prolongation of antibiotic therapy or new indications for source control.

The proportion of embolic events detected in our study is substantially lower than that reported in previous studies, where extracardiac embolic or metastatic lesions were identified in approximately 46–71% of patients undergoing [^18^F]FDG-PET/CT for bacteremia at high risk for IE or other metastatic infection, and newly diagnosed foci in up to 10–48% [16,17,18,19,20,21,22,23]. In our cohort, prior imaging findings were taken into account, and [^18^F]FDG PET/CT showed at least comparable performance to conventional imaging (thoracoabdominal CTA or spine MRI) by detecting all previously identified lesions, while also identifying additional lesions. Several factors may explain the discrepancies between our findings and those reported in most studies. First, because [^18^F]FDG-PET/CT was performed at the discretion of the treating infectious diseases (ID) consultant or the Endocarditis Team, and mainly in diagnostically challenging cases, selection bias may have influenced the results. Second, prior imaging was not consistently reported in most studies, and it was often unclear whether lesions detected by [^18^F]FDG-PET/CT were newly identified or already known at the time of the examination, despite a comparable delay between the first positive blood culture and [^18^F]FDG-PET/CT in our study (median of 8 days) and in previous reports (median ranging from 7 to 11 days) [16,17,18,19,20,21,22,23]. Third, most published studies focused exclusively on patients with complicated *S. aureus* bacteremia, who are at inherently higher risk of embolic complications or bone and joint infections than the broader population of patients with bacteremia of any etiology and suspected IE included in our cohort [17,18,19,21,23,24]. In the only study that took into account prior conventional imaging and included only patients with IE, a new embolic event was found in 29/152 patients (19%), which is comparable to that observed in the present study among patients with IE (16%) [16].

As expected, embolic events were more commonly detected among episodes with IE than among those without, an observation that is consistent with the pathophysiology of septic embolization from endocardial vegetations rather than from other, non-cardiac sources of bacteremia. Embolic events detected on [^18^F]FDG-PET/CT led to reclassification according to the 2023 Duke-ISCVID clinical criteria in only 0.9% of IE episodes. These results raise an important question regarding the clinical value of systematic extracardiac screening for silent embolic lesions in patients with suspected IE, as currently recommended by the ESC guidelines, including in asymptomatic patients [5]. Although [^18^F]FDG-PET/CT, like conventional thoracoabdominal CTA or spine MRI, reliably detects previously unrecognized embolic lesions, the detection of an asymptomatic embolic event rarely changed the diagnostic classification of IE and, on its own, infrequently altered management. This suggests that the diagnostic and therapeutic yield of systematic embolic screening may be more limited than previously assumed, and that detection of a silent embolic lesion does not necessarily translate into a meaningful change in patient outcome [5].

By contrast, the identification of occult, treatable metastatic infectious foci, particularly bone and joint infection and vascular graft infection, was the principal driver of management changes in our cohort. Prolongation of antibiotic therapy or the establishment of a new indication for source control occurred in fewer than 5% of episodes, compared with rates of 15–50% reported in prior studies [16,17,18,21,25]. Several factors likely explain this discrepancy. First, nearly all patients in our cohort were evaluated and followed by ID consultants, ensuring a thorough diagnostic workup, including microbiological assessment and conventional imaging, before [^18^F]FDG-PET/CT was performed, thereby reducing the incremental value of the scan. Notably, restricting the analysis to episodes without prior thoracoabdominal imaging yielded comparable results, suggesting that prior imaging alone does not fully account for the limited impact observed. Second, management changes reported in most prior studies were often driven by both cardiac and extracardiac findings, whereas our analysis intentionally focused on the impact of extracardiac lesions alone [17,18,19,20,21,23]. Douiyeb et al., who also specifically evaluated extracardiac lesions, reported that [^18^F]FDG-PET/CT led to a prolongation of antibiotic treatment in 8 (5%) of 152 patients, which is comparable to that observed in the present study among IE episodes (4%) [16]. In addition, a new indication for source control was reported in 18 of 152 patients (12%), a proportion higher than that observed among IE episodes in the present study (3%) [16]. This discrepancy may partly be explained by differences in patient selection. In the study by Douiyeb et al., patients transferred to other hospitals were excluded, which may have introduced selection bias, as patients remaining in the tertiary-care center were likely those with more severe or complex disease and therefore at higher probability of requiring source control interventions [16].

In the present study, all patients in whom [^18^F]FDG-PET/CT altered management had either bone and joint infections, mainly spondylodiscitis, or extracardiac prosthetic material, such as vascular grafts, consistent with previously shown [16,17,21]. These findings reinforce the notion that the clinical utility of [^18^F]FDG-PET/CT is likely greatest in selected high-risk subgroups, namely bacteremia of unknown source or the presence of vascular prosthetic material, rather than in unselected patients evaluated for suspected IE. Ongoing randomized trials, including the [^18^F]FDG-PET/CT domain of the *S. aureus* Network Adaptive Platform (SNAP) trial, are expected to further clarify the clinical impact of [^18^F]FDG-PET/CT in patients with *S. aureus* bacteremia [26].

This study has several limitations. First, its monocentric, retrospective design over a 10-year period may limit generalizability, and changes in reporting practices, technology, and pre-imaging management over time may have influenced both [^18^F]FDG-PET/CT indications and interpretation. Nevertheless, to our knowledge, this remains the largest study to date evaluating the impact of [^18^F]FDG-PET/CT in patients with bacteremia and suspected IE, and consistent use of the 2023 Duke-ISCVID criteria throughout mitigates this concern. Second, the study is subject to substantial selection bias and confounding by indication, as [^18^F]FDG-PET/CT was performed at the discretion of the responsible ID consultant or Endocarditis Team rather than systematically, and was reserved mainly for diagnostically challenging episodes with uncertain IE or unclear infectious source. This enriches the cohort for higher pre-test probability of complex or atypical infection, and findings should be interpreted as applicable to this selected population. Only 500 of 3033 episodes (16%) with bacteremia and suspected IE underwent [^18^F]FDG-PET/CT (Figure 1); among the 2533 who did not, 1474 (58%) were at low risk for IE and 609 already had a diagnosis established by other cardiac imaging [5]. Accordingly, our findings should be interpreted in the context of this real-world, clinically selected population and should not be directly extrapolated to an unselected population of patients with bacteremia. Nevertheless, the study reflects contemporary clinical practice and provides insight into the potential role of [^18^F]FDG-PET/CT when used as part of the diagnostic work-up of diagnostically challenging bacteremic episodes. Third, use of [^18^F]FDG-PET/CT also increased over time, particularly after the Endocarditis Team was established in 2018, which may have influenced patient selection and diagnostic yield [27]. Fourth, the incremental diagnostic impact of [^18^F]FDG-PET/CT may have been attenuated because conventional imaging, including thoracoabdominal CTA or spine MRI, had already been performed in almost half of the episodes, limiting its incremental yield and direct comparison with these modalities. Fifth, although both cohorts have contributed to previous publications addressing distinct research questions, including the evaluation of IE prediction scores in patients with bacteremia, comparisons of different versions of the Duke criteria, the impact of cerebral and thoracoabdominal imaging on IE diagnosis, the incidence of embolic events in patients with IE, and the impact of the Endocarditis Team on IE outcomes, the present analysis is the first study from these cohorts to specifically evaluate the diagnostic and clinical impact of extracardiac [^18^F]FDG-PET/CT findings using the 2023 Duke-ISCVID criteria [3,4,7,13,24,27,28]. Lastly, since previous studies used earlier versions of the Duke criteria [14,15,16,28], a supplementary analysis using the 2015 Duke-ESC criteria yielded similar results, with only 0.9% of IE cases reclassified based on [^18^F]FDG-PET/CT-detected embolic events.

## 4. Materials and Methods

### 4.1. Study Design

This retrospective study was conducted at Lausanne University Hospital, a Swiss tertiary-care center, and included patients enrolled between January 2015 and September 2025. Patients were drawn from the institutional cohort of patients with suspected IE (January 2015 to September 2025), in which all patients undergo both blood cultures and echocardiography as part of IE work-up, and from the bacteremia cohort (January 2015 to December 2024). The study was approved by the Ethics Committee of the Canton of Vaud (CER-VD 2017-02137, CER-VD 2021-02516).

### 4.2. Study Population

We included adult patients (≥18 years) with bacteremia and suspicion of IE who underwent an [^18^F]FDG-PET/CT examination and had no documented objection to the reuse of their data. Duplicate episodes identified across the two cohorts were excluded. Suspicion of IE was defined by the concomitant performance of blood culture collection and echocardiography specifically to investigate IE.

### 4.3. Definitions

Demographic, clinical, microbiological, and imaging data were manually extracted from electronic health records and verified by an ID specialist. IE diagnosis was established according to the 2023 Duke-ISCVID criteria and included episodes classified as definite or possible IE according to the final adjudication based on the complete set of clinical, pathological, and rejection criteria [6]. The presence of alternative infectious foci was determined by the ID consultant based on clinical, microbiological, radiological, and surgical findings. The decision to perform [^18^F]FDG-PET/CT, as well as its indication, either to assess cardiac metabolic activity in the presence of prosthetic intracardiac material, to detect embolic events and/or metastatic infectious foci, or to detect a primary focus of infection, was made by the responsible ID consultant or by the Endocarditis Team.

Embolic events detected exclusively by [^18^F]FDG-PET/CT were defined as lesions not identified on clinical examination or imaging (thoracoabdominal CT and/or spine MRI) performed prior to the [^18^F]FDG-PET/CT study. Bacteremia was classified as community-acquired, healthcare-associated, or nosocomial according to the criteria proposed by Friedman et al. [29]. Sepsis and septic shock were defined in accordance with the Third International Consensus Definitions for Sepsis and Septic Shock (Sepsis-3) [30]. IE diagnosis was based on the 2023 Duke-ISCVID criteria, and included episodes classified as definite or possible IE according to the final adjudication based on the complete set of clinical, pathological, and rejection criteria [6]. Diagnosis of vascular graft infection was based on the Management of Aortic Graft Infection Collaboration (MAGIC) criteria [31]. Other foci of infection were identified by the responsible ID consultant.

### 4.4. [^18^F]FDG-PET/CT Protocol

To suppress physiological myocardial [^18^F]FDG uptake, patients underwent dietary preparation before the exam. This protocol evolved over the study period: initially, prolonged fasting for >24 h [32]; subsequently, a 72-h high-fat, high-protein, very-low-carbohydrate diet combined with intravenous unfractionated heparin (50 IU/kg, maximum 5000 IU) administered 15 min before [^18^F]FDG administration [33]; or a shorter 24-h low-carbohydrate, high-fat diet combined with 12 h overnight fasting, administration of a high-fat low-carbohydrate beverage and intravenous unfractionated heparin (maximum 30 IU/kg) 4 h before [^18^F]FDG administration [34]. Serum beta-hydroxybyturate was later introduced to assess the success of the dietary preparation in suppressing the physiologic [^18^F]FDG myocardial uptake, using diagnostic threshold as proposed in the literature [35].

Two different PET/CT systems were used for the image acquisition, either a Discovery LS PET/CT (GE HealthCare, Waukesha, WI, USA) or SiPM 3D PET/CT (Biograph Vision 600, Siemens Medical Solutions, Knoxville, TN, USA). PET/CT acquisition was performed 60 min after intravenous administration of 3.5 MBq/kg or 2 MBq/kg of [^18^F]FDG, respectively (depending on the type of scanner), followed by a low-dose CT for attenuation correction. Gated cardiac images were acquired over 10 min, followed by acquisition of whole-body images (from the skull to the feet). Images were reconstructed from the list-mode acquisition using time-of-flight and point-spread function information combined with ordered-subset expectation maximization iterative reconstruction into static images cardiac and whole-body static images. All studies were reviewed by experienced senior nuclear physicians. Adequacy of the suppression of physiological myocardial glucose uptake was assessed and paravalvular pathological uptake was assessed in patients with adequate physiologic myocardial suppression.

### 4.5. Outcomes

The diagnostic impact of [^18^F]FDG-PET/CT included the detection of new embolic events and new infectious foci, and reclassification according to the 2023 Duke-ISCVID clinical criteria [6]. To allow comparison with previous studies, a secondary analysis was performed using the 2015 Duke-ESC clinical criteria [14,15,16,28,36]. As the present study focused specifically on the contribution of extracardiac lesions detected by [18F]FDG-PET/CT, reclassification based on cardiac abnormal metabolic activity, an association already well established in patients with prosthetic intracardiac material [5,14], was not the focus of this analysis and was therefore not systematically re-adjudicated here. The impact of extracardiac findings identified by [^18^F]FDG-PET/CT on clinical management was categorized as either prolongation of antibiotic treatment duration or establishment of a new indication for source control intervention.

### 4.6. Statistical Analysis

Statistical analyses were performed using SPSS version 26.0 (SPSS Inc., Chicago, IL, USA). Continuous variables were analyzed using the Mann–Whitney U test, and categorical variables using the chi-square or Fisher’s exact test, as appropriate. No data were missing in the included variables. All statistical tests were two-tailed, and a *p* < 0.05 was considered statistically significant.

## 5. Conclusions

In conclusion, although [^18^F]FDG-PET/CT can detect embolic events not identified by thoracoabdominal CTA in patients with suspected IE, these findings had limited impact on diagnostic classification, suggesting that systematic use of this modality, or of conventional imaging, solely to screen for peripheral embolic events for diagnostic purposes may not be warranted, and supporting a more selective approach. However, [^18^F]FDG-PET/CT also identified extracardiac infectious foci in a subset of patients, including asymptomatic bone and joint infections and vascular graft infections, with consequent changes in clinical management. Taken together, these findings suggest that the principal clinical value of [^18^F]FDG-PET/CT in suspected IE lies in the detection of treatable occult infectious foci rather than in the systematic screening for silent embolic events, and that this modality may be particularly useful in patients with bacteremia of unknown source or with vascular grafts, in whom the likelihood of occult metastatic or graft-related infection is higher. Future randomized controlled trials are warranted to better define the incremental diagnostic value of [^18^F]FDG-PET/CT over conventional imaging modalities and to determine its impact on patient-centered outcomes.

## Figures and Tables

**Table 1:** Comparison of episodes with or without infective endocarditis diagnosis among 500 episodes with bacteremia and [^18^F]FDG-PET/CT.

Features	No Infective Endocarditis (*n* = 289)	Infective Endocarditis (*n* = 211)	*p* Value
Demographics			
Male sex	214 (74)	152 (72)	0.683
Age (years)	72 (61–79)	72 (62–79)	0.740
Cardiac predisposing factors			
Intravenous drug use	8 (3)	4 (2)	0.572
Congenital disease	10 (4)	17 (8)	0.038
Moderate or severe valve regurgitation/stenosis	54 (19)	36 (17)	0.724
Prior endocarditis	13 (5)	32 (15)	<0.001
Surgical prosthetic valve/transcatheter aortic valve replacement	50 (17)	106 (50)	<0.001
CIED	76 (26)	72 (34)	0.060
Duke minor predisposition criterion	152 (53)	169 (80)	<0.001
Extracardiac prosthetic material			
Arthroplasty	58 (20)	40 (19)	0.820
Osteosynthesis/spondylodesis	16 (6)	6 (3)	0.187
Vascular graft	68 (24)	35 (17)	0.073
Microbiological data			
*S. aureus*	126 (44)	89 (42)	0.784
Coagulase-negative Staphylococci	28 (10)	22 (10)	0.880
Streptococci	47 (16)	33 (16)	0.902
Enterococci	38 (13)	50 (24)	0.003
Gram-positive (other than Staphylococci, Streptococci, Enterococci)	8 (3)	6 (3)	1.000
HACEK	2 (0.7)	4 (2)	0.246
Gram-negative (other than HACEK)	46 (16)	9 (4)	<0.001
Polymicrobial bacteremia	23 (8)	6 (3)	0.019
Duke major microbiological criterion	14 (80)	193 (98)	<0.001
Duke minor microbiological criterion	45 (16)	4 (2)	<0.001
Setting of infection onset			<0.001
Community	99 (34)	138 (65)	
Healthcare-associated	69 (24)	35 (17)	
Nosocomial	121 (42)	38 (18)	
Manifestations			
Duke minor fever criterion	232 (80)	183 (87)	0.070
Sepsis or septic shock	117 (44)	103 (49)	0.318
Duke minor immunologic phenomenon criterion	12 (4)	15 (7)	0.164
Cardiac imaging (other than [^18^F]FDG-PET/CT)			
Transthoracic echocardiography	266 (92)	191 (91)	0.629
Typical lesion ^a^ of IE found on transthoracic echocardiography	0 (0)	32 (15)	<0.001
Transesophageal echocardiography	149 (52)	167 (79)	<0.001
Typical lesion ^a^ of IE found on transesophageal echocardiography	0 (0)	90 (43)	<0.001
Cardiac-CT	19 (7)	17 (8)	0.600
Typical lesion ^a^ of IE found on cardiac CT	0 (0)	12 (6)	<0.001
Thoracoabdominal imaging prior to [^18^F]FDG-PET/CT			
Thoracoabdominal CT	130 (45)	115 (55)	0.038
Spinal MRI	22 (8)	24 (11)	0.161
[^18^F]FDG-PET/CT data			
Time to [^18^F]FDG-PET/CT (days)	8 (4–11)	8 (5–11)	0.684
Adequate suppression of physiological myocardial glucose	228 (79)	178 (84)	0.133
Indications for [^18^F]FDG-PET/CT			
Detection of cardiac lesion in prosthetic intracardiac material	118 (41)	139 (67)	<0.001
Detection of focus of bacteremia	89 (31)	87 (41)	0.018
Detection of embolic event	289 (100)	211 (100)	-
Abnormal cardiac metabolic activity	2 (0.7)	105 (50)	<0.001
Native valve	1 (0.3)	35 (17)	<0.001
Prosthetic valve	1 (0.4)	62 (29)	<0.001
CIED-lead	0 (0)	13 (6)	<0.001
Duke major imaging criterion without [^18^F]FDG-PET/CT results	6 (0.4)	116 (55)	<0.001
Duke major imaging criterion including [^18^F]FDG-PET/CT results	8 (3)	164 (78)	<0.001
Embolic events prior to [^18^F]FDG-PET/CT	21 (7)	56 (27)	<0.001
Cerebral	2 (0.7)	45 (21)	<0.001
Ocular	7 (2)	13 (6)	0.040
Limb	3 (1)	14 (7)	0.001
Thoracoabdominal	9 (3)	14 (7)	0.083
Embolic events detected on [^18^F]FDG-PET/CT	17 (6)	43 (20)	<0.001
Embolic events detected only on [^18^F]FDG-PET/CT	9 (3)	33 (16)	<0.001
Duke minor vascular phenomenon criterion before [^18^F]FDG-PET/CT	21 (7)	56 (27)	<0.001
Duke minor vascular phenomenon criterion including [^18^F]FDG-PET/CT results	27 (9)	77 (37)	<0.001
Infectious focus other than IE detected only on [^18^F]FDG-PET/CT	20 (7)	11 (5)	0.460
BJI detected only on [^18^F]FDG-PET/CT	10 (4)	8 (4)	1.000
Vascular graft infection detected only on [^18^F]FDG-PET/CT	10 (4)	3 (1)	0.254
Malignancy	5 (2)	4 (2)	1.000
Classification according to 2023 Duke-ISCVID clinical criteria without [^18^F]FDG-PET/CT extracardiac findings			<0.001
Rejected	84 (29)	2 (0.9)	
Possible	193 (67)	51 (24)	
Definite	12 (4)	58 (75)	
Classification according to 2023 Duke-ISCVID clinical criteria including [^18^F]FDG-PET/CT extracardiac findings			<0.001
Rejected	84 (29)	2 (0.9)	
Possible	190 (66)	49 (23)	
Definite	15 (5)	160 (76)	
Modifications of management due to [^18^F]FDG-PET/CT extracardiac findings			
Prolongation of antibiotic treatment duration	20 (7)	8 (4)	0.168
New indication for source control intervention	16 (6)	7 (3)	0.285

Data are presented as numbers (%) or median (interquartile range). ^a^ Vegetation, perforation, abscess, aneurysm, pseudoaneurysm, fistula. [^18^F]FDG-PET/CT: [^18^F]Fluorodeoxyglucose Positron Emission Tomography/Computed Tomography; BJI: bone and joint infection; CIED: cardiac implantable electronic device; IE: infective endocarditis; ISCVID: International Society for Cardiovascular Infectious Diseases.

**Table 2:** Types of embolic events and metastatic infections identified by [^18^F]FDG-PET/CT.

Features	Patients without Prior Thoracoabdominal CT (*n* = 255)	Patients with Prior Thoracoabdominal CT (*n* = 245)		
	Lesions Identified by [^18^F]FDG-PET/CT	Lesions Identified by Thoracoabdominal CT and/or Spine MRI	Lesions Identified by [^18^F]FDG-PET/CT^a^	Lesions Identified Only by [^18^F]FDG-PET/CT
Embolic events	18 (7)	22 (9)	42 (17)	24 (10)
Pulmonary	12 (5)	13 (5)	25 (10)	12 (5)
Splenic	2 (0.8)	9 (4)	17 (7)	8 (3)
Renal	2 (0.8)	5 (2)	7 (3)	2 (0.8)
Hepatic	1 (0.4)	0 (0)	3 (1)	3 (1)
Mycotic aneurysm	1 (0.4)	1 (0.4)	3 (1)	2 (0.8)
Major arterial emboli	1 (0.4)	0 (0)	1 (0.4)	1 (0.4)
Metastatic infections				
Vascular graft infection	3 (1)	6 (2)	16 (7)	10 (4)
Bone and joint infection	30 (12) ^b^	36 (15)	43 (18)	8 (3)
Septic arthritis	8 (3) ^b^	8 (3)	9 (4)	1 (0.4)
Spondylodiscitis	18 (7) ^b^	22 (9)	27 (11)	5 (2)
Non-vertebral osteomyelitis	0 (0)	2 (0.8)	2 (0.8)	0 (0)
Prosthetic joint infection	0 (0)	5 (2)	6 (2)	1 (0.4)
Osteosynthesis/spondylodesis infection	2 (0.8) ^b^	2 (0.8)	3 (1)	1 (0.4)

Data are presented as numbers (%). ^a^ all lesions identified by prior thoracoabdominal CT or spine MRI were confirmed by subsequent [^18^F]FDG-PET/CT. ^b^ 4 septic arthritis, 13 spondylodiscitis and 1 spondylodesis infection were detected by spine MRI performed before [^18^F]FDG-PET/CT.

## Data Availability

The data that support the findings of this study are available from the corresponding author upon reasonable request.

## Supplementary Materials

The following supporting information can be downloaded at: https://www.mdpi.com/article/10.3390/antibiotics15100994/s1, Table S1: Classification of 500 episodes with bacteremia and [^18^F]FDG-PET/CT based on the 2015 ESC Duke clinical criteria with and without extracardiac findings of [^18^F]FDG-PET/CT; Table S2: Impact of extracardiac findings of [^18^F]FDG-PET/CT on management.
