# Supplementary material for: Impact of Extracardiac [18F]FDG-PET/CT Findings on Diagnosis and Management in Patients with Suspected Infective Endocarditis
Source: Antibiotics (Basel). 2026 Oct 7;15(10):994. doi: 10.3390/antibiotics15100994 (PMC13700727; doi:10.3390/antibiotics15100994)
Supplement: Supplementary file 1 [file antibiotics-15-00994-s001.zip › antibiotics-4585381-supplementary.pdf]

Table S1. Classification of 500 episodes with bacteremia and [18F]FDG-PET/CT based on the 2015 ESC Duke clinical criteria with and without extracardiac findings of [18F]FDG-PET/CT

**Features**	**No infective endocarditis (*n* = 289)**	**Infective endocarditis (*n* = 211)**	***p* value**
Major criteria			
Microbiological	129 (45)	144 (68)	<0.001
Imaging	3 (1)	138 (65)	<0.001
Minor criteria			
Predisposition	57 (20)	106 (50)	<0.001
Fever	232 (80)	183 (87)	0.070
Immunologic phenomena	12 (4)	15 (7)	0.164
Microbiological	65 (23)	24 (11)	0.001
Vascular phenomena before [18F]FDG-PET/CT	21 (7)	56 (27)	<0.001
Vascular phenomena including [18F]FDG-PET/CT results	27 (9)	77 (37)	<0.001
Classification according to 2015 Duke-ESC clinical criteria without [18F]FDG-PET/CT extracardiac findings			<0.001
Rejected	91 (32)	2 (0.9)	
Possible	189 (65)	54 (25)	
Definite	9 (3)	156 (74)	
Classification according to 2015 Duke-ESC clinical criteria including [18F]FDG-PET/CT extracardiac findings			<0.001
Rejected	90 (31)	2 (0.9)	
Possible	183 (63)	51 (24)	
Definite	16 (6)	158 (75)	

Data appear as numbers (%)

[18F]FDG-PET/CT: [18F]Fluorodeoxyglucose Positron Emission Tomography/Computed Tomography; ESC: European Society of Cardiology

Table S2. Impact of extracardiac findings of [18F]FDG-PET/CT on management

Patient	IE	Infectious foci found on [18F]FDG-PET/CT					Changes on management	
		Septic arthritis	Spondylodiscitis	Prosthetic joint infection	Osteosynthesis or spondylodesis infection	Vascular graft infection	Antibiotic treatment	Indication for source control intervention
1	Y		Y				Prolongation at 6 weeks	CT-guided percutaneous drainage of paravertebral abscess
2	Y			Y			Prolongation at 12 weeks	Debridement, antibiotic, and implant retention
3	Y		Y				Prolongation at 6 weeks	
4			Y				Prolongation at 6 weeks	
5	Y		Y				Prolongation at 6 weeks	CT-guided percutaneous drainage of paravertebral abscess
6	Y		Y					
7	Y		Y				Prolongation at 6 weeks	
8	Y		Y					
9			Y				Prolongation at 6 weeks	CT-guided percutaneous drainage of paravertebral abscess
10			Y				Prolongation at 6 weeks	
11			Y				Prolongation at 6 weeks	
12					Y		Prolongation at 12 weeks	Debridement, antibiotic, and implant retention
13	Y	Y						Arthroscopic lavage
14		Y					Prolongation at 4 weeks	Arthroscopic lavage
15		Y					Prolongation at 4 weeks	
16					Y		Prolongation at 12 weeks	Debridement, antibiotic, and implant retention
17		Y					Prolongation at 4 weeks	Arthroscopic lavage
18		Y					Prolongation at 4 weeks	Arthroscopic lavage
19	Y					Y	Prolongation at 12 weeks	Vascular graft debridement/replacement
20						Y	Prolongation at 12 weeks	Vascular graft debridement/replacement
21	Y					Y	Prolongation at 12 weeks	Vascular graft debridement/replacement
22	Y					Y	Prolongation at 12 weeks	Vascular graft debridement/replacement
23						Y	Prolongation at 12 weeks	Vascular graft debridement/replacement
24						Y	Prolongation at 12 weeks	Vascular graft debridement/replacement
25						Y	Prolongation at 12 weeks	Vascular graft debridement/replacement
26						Y	Prolongation at 12 weeks	Vascular graft debridement/replacement
27						Y	Prolongation at 12 weeks	Vascular graft debridement/replacement

28	Y	Prolongation at 12 weeks	Vascular graft debridement/replacement
29	Y	Prolongation at 12 weeks	Vascular graft debridement/replacement
30	Y	Prolongation at 12 weeks	Vascular graft debridement/replacement
31	Y	Prolongation at 12 weeks	Vascular graft debridement/replacement

[^{18}F]FDG-PET/CT: [^{18}F]Fluorodeoxyglucose Positron Emission Tomography/Computed Tomography; Y: yes
